# Hydrophobization of Reduced Graphene Oxide Aerogel Using Soy Wax to Improve Sorption Properties

**DOI:** 10.3390/ma17112538

**Published:** 2024-05-24

**Authors:** Sergey A. Baskakov, Yulia V. Baskakova, Eugene N. Kabachkov, Mikhail V. Zhidkov, Anastasia V. Alperovich, Svetlana S. Krasnikova, Dmitrii A. Chernyaev, Yury M. Shulga, Gennady L. Gutsev

**Affiliations:** 1 Federal Research Center of Problem of Chemical Physics and Medicinal Chemistry, Russian Academy of Sciences, 142432 Chernogolovka, Moscow Region, Russia; baskakov@icp.ac.ru (S.A.B.); ybaskakova@yandex.ru (Y.V.B.); en.kabachkov@gmail.com (E.N.K.); zhidkov@icp.ac.ru (M.V.Z.); ggrafinovny@gmail.com (A.V.A.); skras27@mail.ru (S.S.K.); chernyayevda@icp.ac.ru (D.A.C.); yshulga@gmail.com (Y.M.S.); 2 Osipyan Institute of Solid State Physics RAS, 142432 Chernogolovka, Moscow Region, Russia; 3 Department of Physics, Florida A&M University, Tallahassee, FL 32307, USA

**Keywords:** graphene oxide, soy wax, composite aerogel, swelling effect, sorption capacity, petroleum products

## Abstract

A special technique has been developed for producing a composite aerogel which consists of graphene oxide and soy wax (GO/wax). The reduction of graphene oxide was carried out by the stepwise heating of this aerogel to 250 °C. The aerogel obtained in the process of the stepwise thermal treatment of rGO/wax was studied by IR and Raman spectroscopy, scanning electron microscopy, and thermogravimetry. The heat treatment led to an increase in the wax fraction accompanied by an increase in the contact angle of the rGO/wax aerogel surface from 136.2 °C to 142.4 °C. The SEM analysis has shown that the spatial structure of the aerogel was formed by sheets of graphene oxide, while the wax formed rather large (200–1000 nm) clumps in the folds of graphene oxide sheets and small (several nm) deposits on the flat surface of the sheets. The sorption properties of the rGO/wax aerogel were studied with respect to eight solvent, oil, and petroleum products, and it was found that dichlorobenzene (85.8 g/g) and hexane (41.9 g/g) had the maximum and minimum sorption capacities, respectively. In the case of oil and petroleum products, the indicators were in the range of 52–63 g/g. The rGO/wax aerogel was found to be highly resistant to sorption–desorption cycles. The cyclic tests also revealed a swelling effect that occurred differently for different parts of the aerogel.

## 1. Introduction

The lotus leaf is commonly considered as an example of the relationship between hydrophobicity and surface morphology [1], sometimes without indicating that the surface of the lotus leaf (*Nelumbo nucifera*) is a composite of papillary epidermal cells and tubular epicuticular waxes [2,3]. Plant waxes can be seen with the naked eye on the leaves and fruits of certain plants. Some plant waxes have found practical uses, for example, carnauba wax extracted from the leaves of the palm tree Copernicia cerifera. This wax has found a wide range of applications, particularly in high-gloss finishes for automobiles and controlled-release pharmaceutical formulations [4].

This work is related to a research direction that can be described as the modification of rough structures, e.g., those of aerogels, with materials possessing low surface energies such as waxes. In particular, we are interested in the possibility of the additional hydrophobization of partially reduced graphene oxide aerogels using wax in order to improve its sorption properties with respect to organic solvents and oil. In principle, rGO/wax composites are known [5,6,7,8]. Wax has often been used as a matrix to form thin samples that effectively absorb electromagnetic waves over a wide bandwidth, where rGO is a part of the filler. In this respect, [8] should be noted, where the dielectric losses of an rGO aerogel were optimized by adding wax. However, we have found no single publication which is devoted to regulating the hydrophilic–hydrophobic properties of aerogels by wax additives.

Previously, we described a method for preparing composite superhydrophobic rGO-polytetrafluoroethylene (PTFE) aerogels and found that isopropanol, acetone, and hexane almost completely filled in the free volumes of the aerogel [9]. The aerogel was also demonstrated to be highly resistant to cyclic loading with solvents. In this work, we describe the preparation of an rGO/wax composite aerogel using soy wax. The aerogel obtained was tested as a sorbent for such solvents as tetrahydrofuran, toluene, acetone, dichlorobenzene, n-hexane, n-heptane, methylpyrrolidone, and propanol-2, as well as crude oil and several petroleum products such as white spirit, kerosene, and machine oil. The sorption capacity of rGO/wax with respect to these solvents turned out to be higher than that of rGO/PTFE, which served as the basis for the certification of the resulting aerogel using various physicochemical methods.

## 2. Experimental Section

### 2.1. Materials

Soy wax (SW) for container candles was purchased from the Candles Only online store (Russia). Note that soy wax is hydrogenated soybean oil composed primarily of unsaturated fatty acids such as linoleic and linolenic acids. A method for obtaining oil from soybeans in described elsewhere [10]. In addition to oil, soybeans also contain many other useful substances [11,12,13,14]. Nonionic surfactant Polysorbate 80 (TWIN 80) was purchased from the Mendeleev Shop online store. We used a modified Hammers method [15] and obtained graphite oxide (GO). The details of our graphene oxide production are described in refs [16,17]. The lateral size of graphene oxide particles was 0.5–5 microns, and the particle thickness was from 0.7 to 1.7 nm.

### 2.2. Synthesis of the rGO/Wax Aerogel

The SW suspension was prepared according to the “oil in water” type; namely, 21 mL of water, 3 g of soy wax, and 0.5 mL of TWIN 80 were added to a glass with a volume of 50 mL. The mixture was thermostatted at 55 °C until the wax was completely dissolved and then dispersed using an ultrasonic homogenizer, MEF 93.1 (MELFIZ-ultrasvuk LLC, Moscow, Russia), until a milky white emulsion was obtained. The procedure for preparing GO/wax aerogels was as follows: 150 mL of a graphene oxide suspension with a concentration of 10 mg/mL was treated with ultrasound for 3 min, and 15 mL of SV emulsion was introduced dropwise into it without stopping the ultrasound exposure. Ultrasound treatment was continued for another 5 min after stopping the injection of the SW emulsion. The resulting dispersion was frozen in cylindrical molds with a volume of 5 mL and mounted on a copper plate cooled with liquid nitrogen.

**Figure 1:**
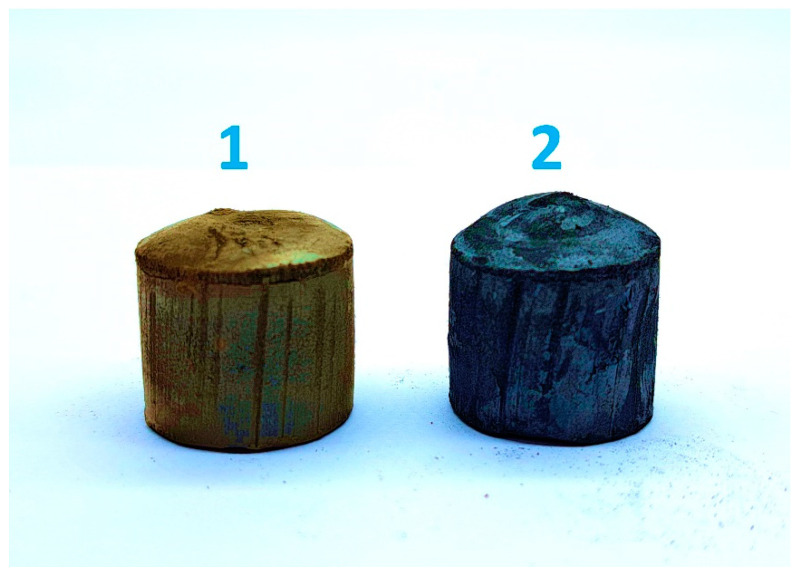
Photograph of aerogel samples: (**1**) is GO/wax and (**2**) is rGO/wax.

The hydrogels were removed from the molds after freezing and were freeze-dried in an IlShin BioBase FD5512 freeze-dryer (Seoul, Republic of Korea). The resulting GO/wax aerogels had a density of 20 ± 2 mg/cm^3^. The aerogels obtained after drying were subjected to heat treatment to remove surfactants and reduce graphene oxide. Heat treatment was carried out stepwise up to 250 °C. The sample was first heated to 100 °C and held for 1 h; then, the temperature was raised by 50 °C at intervals of 1 h. As a result of the treatment, the aerogels changed color from light gray with a brown tint to almost black (Figure 1). The aerogel density after reduction decreased to 13 ± 1 mg/cm^3^. Similar annealing of pure wax led to its melting, but after cooling to room temperature, its color and density remained the same as before melting.

### 2.3. Characterization and Measurements

The IR spectra (resolution 1 cm^−1^, number of scans 32) were recorded at room temperature in the range of 450–4000 cm^−1^ on a Perkin-Elmer “Spectrum Two” Fourier-transform IR spectrometer (Waltham, MA, USA) with an ATR attachment. The Raman spectra were obtained on a Confotec NR500 Raman microscope (SOLinstruments, Minsk, Belarus). The laser excitation wavelength was 532 nm, the power at the measurement point was 0.1 mW, the beam diameter was ~2 µm.

The thermogravimetric analysis (TGA) of the samples was performed using an STA 449 F3 Jupiter instrument (Selb, Bavaria, Germany). The measurements were carried out in the temperature range of 20–550 °C at a rate of 10 °C/min and in a He flow of 50 mL/min. The contact water-wetting angle was measured by using an OCA 20 instrument (Data Physics Instruments GmbH, Filderstadt, Germany) at room temperature. Electron micrographs were obtained on a COXEM EM-30 scanning electron microscope (electron energy of 15 kV and the chamber pressure of 2 × 10^−5^ Pa). The XPS spectra were obtained using a Specs PHOIBOS 150 MCD electron spectrometer (Specs, Berlin, Germany) and an X-ray tube with an Mg anode (hν = 1253.6 eV). The vacuum in the spectrometer chamber did not exceed 4 × 10^−8^ Pa.

### 2.4. Study of Sorption Properties

The capacity of rGO/wax aerogel sorption with respect to organic solvents, crude oil, and petroleum products was studied under static conditions. For this purpose, 100 mL of a solvent, crude oil, or a petroleum product was poured into the test container in such a way that the thickness of a layer of the solvent, crude oil, or petroleum product was at least 2.5 cm.

A pre-weighed aerogel with the mass of 0.070–0.078 g was placed in a mesh basket, which was immersed into a container so that the basket with the aerogel was freely placed inside the container. After 10 min, the basket with the aerogel was removed and the remaining solvent was drained for 1 min. After this, the contents from the basket were transferred to a tray with a known weight. Then, the tray with the aerogel was weighed, and the result was recorded. Sorption capacity (Q_w_) was calculated using the following formula:

Q_w_ = m_s_/m_a_ (1)

where m_s_ is the mass of sorbed solvent, oil, or petroleum product in g and m_a_ is the mass of the original aerogel in g. The volume fraction (%) occupied by solvent, oil, or petroleum products was calculated using the following formula:

Q_v_ = V_a_/V_o_ × 100% (2)

where V_o_ is the volume of dry aerogel and V_a_ is the volume of solvent adsorbed by the aerogel.

Cyclic tests of a sample of rGO/wax aerogel were also carried out in the “sorption–desorption” mode using hexane as an example to assess the possibility of multiple reuses of such a sorbent. To do this, the sample was weighed dry, then soaked in a solvent according to the method described above and weighed again. Next, the sample was dried in an oven at T = 65 °C for one hour. This treatment was sufficient to completely remove the solvent from the aerogel sample. After drying, the sample was weighed again. This set of procedures was considered as one sorption–desorption cycle. A total of 10 such cycles were carried out.

## 3. Results and Discussion

### 3.1. IR Spectra

**Figure 2:**
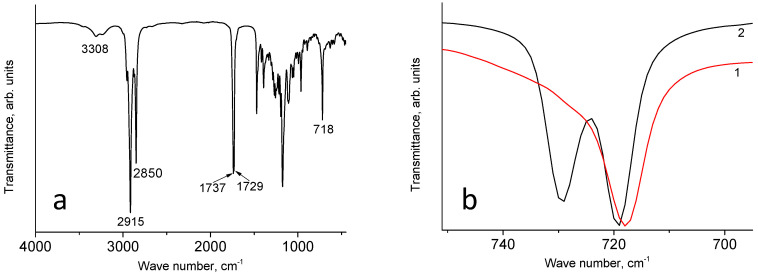
(**a**) IR spectrum of soy wax; (**b**) fragments of the IR spectra of soy wax (1) and paraffin (2).

Let us note that no peaks with noticeable intensity in the region of 3000–3020 cm^−1^ corresponding to the stretching vibrations of =C-H bonds in linoleic acid [18] were detected in the IR spectrum of the wax sample presented in Figure 2a. In the IR spectrum of this wax sample, the most intense peaks correspond to the stretching vibrations of C-H bonds, characteristic of paraffins. However, in the region of the rocking vibrations of methylene groups, there is one peak at 718 cm^−1^ (Figure 2b). The absence of a second peak due to splitting indicates that the alkyl chains in the sample are arranged in a random manner [19].

Another interesting distinctive feature of the spectrum of soy wax with respect to the paraffin spectrum is the presence of a rather intense absorption band in the region of stretching vibrations of O-H bonds with the maxima at 3460 cm^−1^, 3308 cm^−1^ (marked in Figure 2a), and 3234 cm^−1^. Their positions indicate that the OH groups are also connected by hydrogen bonds. There are no peaks with noticeable intensity in the region of 1630 cm^−1^, which is indicative of the absence or extremely low concentration of water molecules in soy wax. A fairly intense absorption band, corresponding to the stretching vibrations of C=O bonds, has two maxima at 1737 cm^−1^ and 1729 cm^−1^. In terms of the ν(C=O) value, our sample of soy wax is different from the samples of soy wax described previously, where only a single peak was observed at 1745 cm^−1^ [20] and 1744 cm^−1^ [21] in this spectral region.

The IR spectrum of graphene oxide is well known, and the IR spectrum of the graphene oxide used in this work is shown in Figure S1. In accordance with the literature data [22,23,24], the absorption bands in the range of 3000–3700 cm^−1^ can be attributed to vibrations of O–H bonds. The bands at 2919 cm^−1^ and 2850 cm^−1^ are apparently due to vibrations of C–H bonds belonging to aliphatic groups of carbon-based impurities of the sample which was stored in air. The band at 1733 cm^−1^ can be attributed to vibrations of C=O bonds of carbonyl groups or ketones. The band at 1620 cm^−1^ is probably composite and may contain contributions from both vibrations of double C=C bonds [25] and deformation vibrations of water molecules. The band at 1162 cm^−1^ is attributed to vibrations of C–OH bonds [26], and the band at 1046 cm^−1^ belongs to the vibrations of C–O bonds from different groups, including alcoxy and epoxy groups [22]. Other authors also observed a band at 877 cm^−1^ (see, e.g., Ref. [27]); however, no assignment of the band was conducted. It is highly likely that this peak belongs to deformation vibrations of epoxy groups.

**Figure 3:**
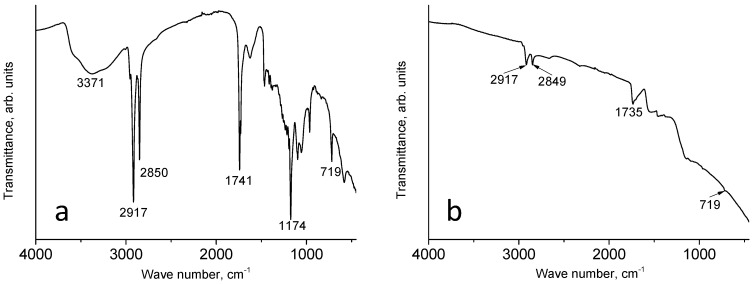
IR spectra GO/wax (**a**) and rGO/wax (**b**).

Characteristic features of both components can be seen in the IR spectrum of the GO/wax aerogel in Figure 3a. A wide intense peak with a maximum at 3371 cm^−1^ is due to stretching vibrations of O-H bonds of GO. The spectrum of this aerogel also shows the characteristic numerous low-intensity narrow peaks in the wax “fingerprint” region. It is possible that the spectrum of the GO/wax aerogel cannot be described by a simple sum of the spectra of GO and wax due to the presence of small amounts of surfactants and transformations that may take place during the synthesis and drying of the GO/wax aerogel. Such an analysis is beyond the scope of this work.

It is interesting to analyze the spectrum of the aerogel after restorative heat treatment, i.e., rGO/wax as the target product (see Figure 3b). First of all, one can note in the spectrum of rGO/wax the inclined background, the absence of a broad band of stretching vibrations of O-H bonds, and the characteristic numerous narrow peaks in the “fingerprint” region of the wax. Such changes with respect to the GO/wax IR spectrum indicate a high degree of graphene oxide reduction. A slanted background usually appears in conductive rGO-based samples [28]. Despite the absence of characteristic narrow low-intensity peaks, the positions of the most intense wax peaks at 2917 cm^−1^, 2835 cm^−1^, 1736 cm^−1^, and 719 cm^−1^ were preserved. One may assume that the wax is shielded by the conductive rGO sheets.

### 3.2. Raman Spectra

**Figure 4:**
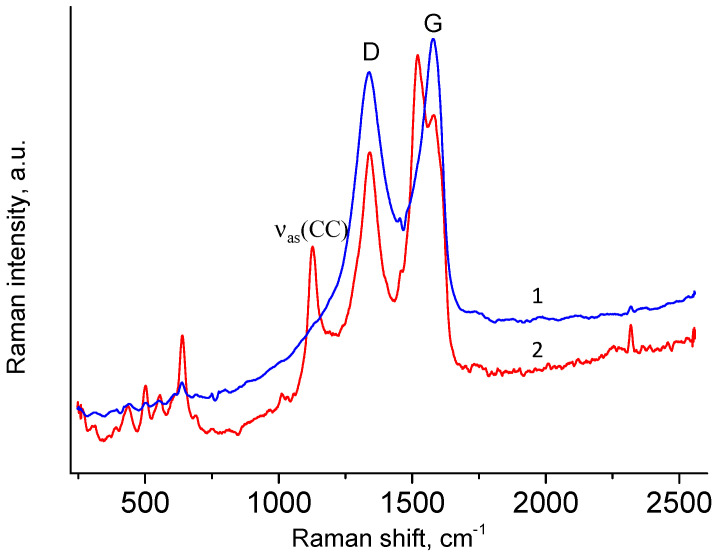
Raman spectra of GO/wax aerogel in two different points.

Figure 4 shows the Raman spectra obtained at different points of the rGO/wax aerogel. It can be seen that the main contribution to spectrum 1 displayed in blue color is due to the peaks with maxima at 1337 cm^−1^ and 1578 cm^−1^. The positions of these peaks coincide with the positions of peaks D and G in the spectrum of graphene oxide [29,30,31,32,33]. In addition to peaks D and G, other narrower peaks can be seen in spectrum 2, displayed in red color. The positions of some peaks are the same as peak positions in the paraffin spectra; in particular, the position of the peak at 1128 cm^−1^ coincides with the position of the ν_as_(CC) peak of paraffin [34,35,36,37]. It can be thought that this point of the analysis zone contains mostly soy wax. Thus, the Raman method indicates a nonuniform distribution of components in the aerogel under study.

### 3.3. SEM Images

**Figure 5:**
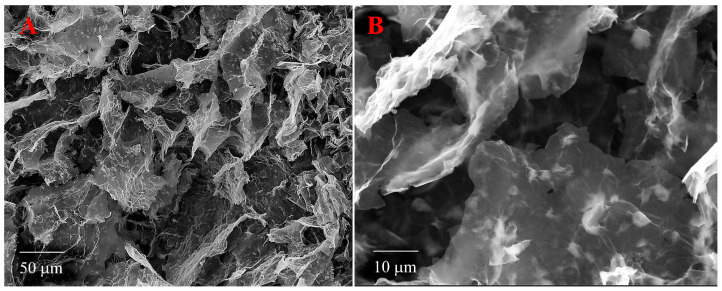
Two SEM images of rGO/wax with different resolutions of 50 μm (**A**) and 10 μm (**B**).

Two SEM images of the rGO/wax aerogel are presented in Figure 5. It can be seen that the spatial structure with large, interconnected voids is formed by sheets of graphene oxide. The wax forms both rather large (200–1000 nm) clumps in the folds of graphene oxide sheets and small (several nm) deposits on the flat surface of these sheets. In the case of the rGO/PTFE (PTFE stands for polytetrafluoroethylene) aerogel, such homogeneity was not observed [9].

### 3.4. TGA Analysis

**Figure 6:**
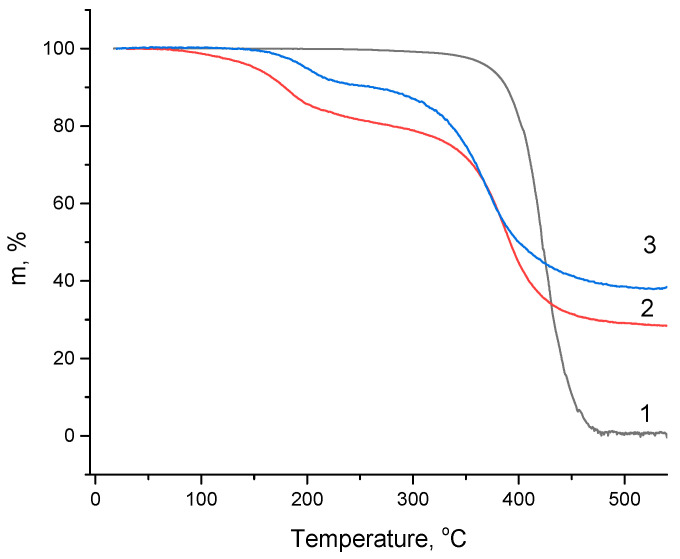
TGA curves for wax (1), GO/wax (2), and rGO/wax (3).

Figure 6 shows TGA curves for wax along with the GO/wax and rGO/wax composites. One can state that the presence of GO or rGO leads to losses beginning at 100 °C (GO) and 150 °C (rGO), which could be associated with the release of water from these components. It can be seen that the water loss in the case of graphene oxide is greater than in the case of reduced graphene oxide. For wax, the maximum rate of weight loss and complete weight loss occurs at 420 °C and 470 °C, respectively. At 550 °C, the weight losses of the GO/wax and rGO/wax samples are 71.6 and 49.3%, respectively.

Interestingly, the maximum loss rates of the GO/wax and rGO/wax composites were observed at temperatures of 388 °C and 371 °C, respectively, and these losses can be associated with wax evaporation (the samples were heated in a stream of high-purity helium). The second interesting fact that follows from our TGA studies is that the amount of evaporated wax, calculated from the TGA curves for rGO/wax, of 52% is slightly higher than that of GO/wax (50%). This means that during the reduction of graphene oxide at 250 °C, its weight fraction decreases due to the release of water.

### 3.5. CWA Results

The contact wetting angle (CWA) for the flat soy wax surface was found to be 100.5°. In the case of the GO/wax aerogel obtained in the shape of a cylinder (Figure 1), the CWA value measured for a flat end surface is significantly higher and equals 136.2°. The annealing procedure increases the contact angle of the end surface to 142.4°. Let us note that a superhydrophobic graphene aerogel with CWA = 151.1–153.9° was previously obtained by using surface chemical reduction [38]. For the rGO/PTFE aerogel we obtained, the CWA value was in the range of 161.9–163.7° [9]. In this regard, the rGO/wax samples obtained in this work are not outstanding.

### 3.6. Adsorption Properties

The sorption properties of rGO/wax aerogel were studied in relation to a large set of solvents with different chemical compositions, including water, oil, and petroleum products such as white spirit, kerosene, and machine oil. The data obtained are presented in Table 1, where the data on the sorption properties of the rGO/PTFE composite aerogel [9] are shown for comparison.

It could be seen that the rGO/wax aerogel is superior to the rGO/PTFE aerogel in the Q_w_ and Q_v_ parameters defined in Equations (1) and (2), respectively, whereas the opposite was found with respect to the CWA values. From this, it follows that a high contact angle with respect to water does not always imply a high sorption level for hydrophobic sorbates. The superiority of rGO/wax aerogel over rGO/PTFE aerogel is also reflected in its easier and more environmentally friendly production process. We note that the Q_v_ value of 101.8 for propanol-2 is not an error as it has been verified several times. Apparently, this is a consequence of the swelling of the aerogel.

**Figure 7:**
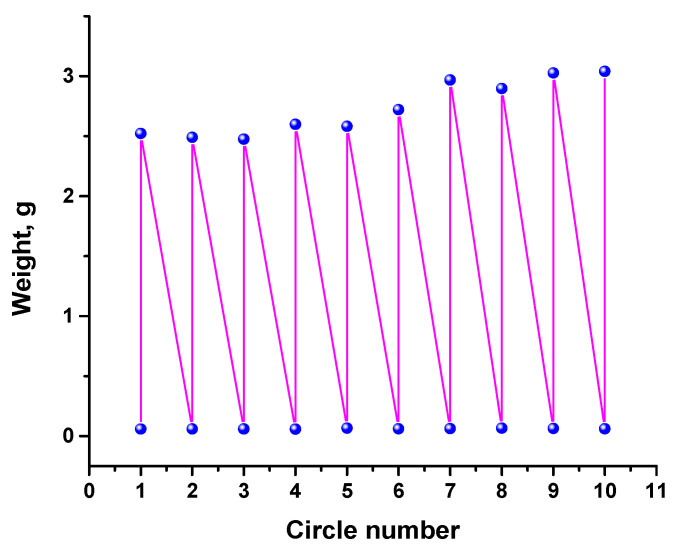
Sorption recyclability of the aerogel rGO/wax for hexane.

The stability of rGO/wax aerogel to sorption–desorption cycles was tested using hexane as an example. The test results are shown in Figure 7, from where it follows that the aerogel is highly resistant to cyclic loading with a solvent. Moreover, its capacity after the third cycle increased by 10–15%, which means that the sample swells during cycling, i.e., its volume increases.

**Figure 8:**
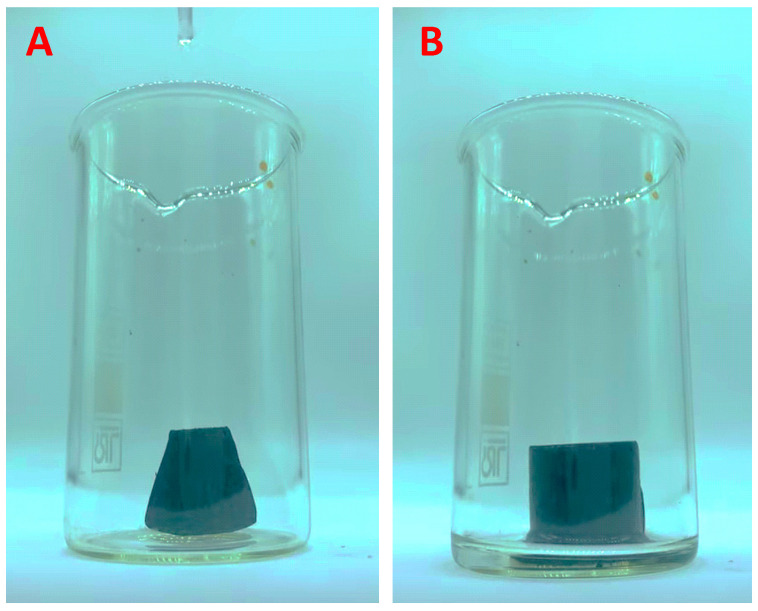
Photographs of dry rGO/wax aerogel (**A**) and rGO/wax aerogel impregnated with hexane (**B**).

During cyclic tests, it was observed that swelling during the sorption of hexane occurs differently for different parts of the dry rGO/wax aerogel, as shown in Figure 8. A video of the swelling process during hexane sorption can be seen in the Supplementary Information. After the desorption of hexane, significant shrinkage of the sample along one of the bases of the cylinder was observed, and the sample again became similar to a truncated cone. These shape changes occurred in each of the 10 cycles of sorption tests. We associate changes in the shape of the aerogel during the sorption–desorption cycles with the peculiarities of the synthesis, in particular with the temperature gradient at the freezing stage before freeze-drying.

## 4. Conclusions

In this work, a method for producing a composite aerogel consisting of graphene oxide and soy wax (GO/wax) was developed. The reduction of graphene oxide was carried out by the stepwise heating of this aerogel to 250 °C. The resulting rGO/wax aerogel was studied by IR and Raman spectroscopy, scanning electron microscopy, and thermogravimetry. It was found that annealing to 250 °C increases the proportion of wax in the composite aerogel. Also, the contact angle of the flat base surface of the rGO/wax aerogel obtained in the form of cylinder increases from 136.2° to 142.4°.

By means of scanning electron microscopy, it was shown that the spatial structure of the aerogel is formed by sheets of graphene oxide. The wax forms both rather large (200–1000 nm) clumps in the folds of graphene oxide sheets and small (several nm) deposits on the flat surface of these sheets. The sorption properties of the rGO/wax aerogel were studied in relation to eight solvents with different chemical compositions, namely, tetrahydrofuran; toluene; acetone; dichlorobenzene; n-hexane; n-heptane; methylpyrrolidone; propanol-2; crude oil; and petroleum products such as white spirit, kerosene, and machine oil.

Among the tested solvents, the maximum and minimum sorption capacities were shown by dichlorobenzene (85.8 g/g) and hexane (41.9 g/g), respectively. In the case of crude oil and petroleum products, these indicators are in the range of 52–63 g/g. The rGO/wax aerogel was found to be highly resistant to sorption–desorption cycles. In the case of hexane, the sorption capacity even increases by 10–15% after the third cycle. The cyclic tests also revealed a swelling effect that occurs differently for different parts of the aerogel.

## Figures and Tables

**Table 1:** The aerogel sorption properties.

	Sorbate	Q_w_ ^a^, g/g		Q_v_ ^a^, %	
		rGO/Wax	rGO/PTFE ^b^	rGO/Wax	rGO/PTFE ^b^
1	Tetrahydrofuran	64.38	23.23	99.91	82.9
2	Toluene	60.70	15.52	96.62	56.6
3	Acetone	52.31	23.15	91.39	94.1
4	Dichlorobenzene	85.83	0.25	91.12	6.20
5	N-hexane	41.86	18.97	87.99	92.5
6	N-heptane	48.58	-	98.68	-
7	Methylpyrrolidone	69.40	-	92.98	-
8	Propanol-2	57.94	23.61	101.80	95.9
9	Petroleum	63.11	-	97.85	-
10	Kerosene	53.87	-	87.46	-
11	White spirit	52.43	-	91.59	-
12	Machine oil	56.55	-	91.81	-
13	Water	0.05	-	0.06	-

^a^ The Q_w_ and Q_v_ parameters are defined by Equations (1) and (2); ^b^ the data from Ref. [9].

## Data Availability

The original contributions presented in the study are included in the article, further inquiries can be directed to the corresponding author.

## Supplementary Materials

The following supporting information can be downloaded at: https://www.mdpi.com/article/10.3390/ma17112538/s1, Figure S1: The IR spectrum of GO; Table S1: Positions of the main peaks in the IR spectrum of soy wax; Video S1: Change in aerogel shape (swelling effect) upon solvent adsorption.
