# Supplementary material for: Hydrophobization of Reduced Graphene Oxide Aerogel Using Soy Wax to Improve Sorption Properties
Source: Materials (Basel). 2024 May 24;17(11):2538. doi: 10.3390/ma17112538 (PMC11174041; doi:10.3390/ma17112538)
Supplement: Supplementary file 1 [file materials-17-02538-s001.zip › SI_Figure and table.pdf]

Hydrophobization of reduced graphene oxide aerogel using soy wax to improve sorption properties

Sergey A. Baskakov [1] Yulia V. Baskakova [1], Eugene N. Kabachkov [1.2], Mikhail V. Zhidkov [1], Anastasia V. Alperovich [1]. Svetlana S. Krasnikova [1], Dmitrii A. Chernyaev [1]. Yury M. Shulga [1] and Gennady L. Gutsev[3]*

[1] Federal Research Center of Problem of Chemical Physics and Medicinal Chemistry. Russian Academy of Sciences. Chernogolovka. 142432 Moscow Region. Russia
[2] Osipyan Institute of Solid State Physics RAS. Moscow Region. Chernogolovka 142432. Russia
[2] Department of Physics. Florida A&M University. Tallahassee. FL 32307. USA

Video S1: Change in aerogel shape (swelling effect) upon solvent adsorption.

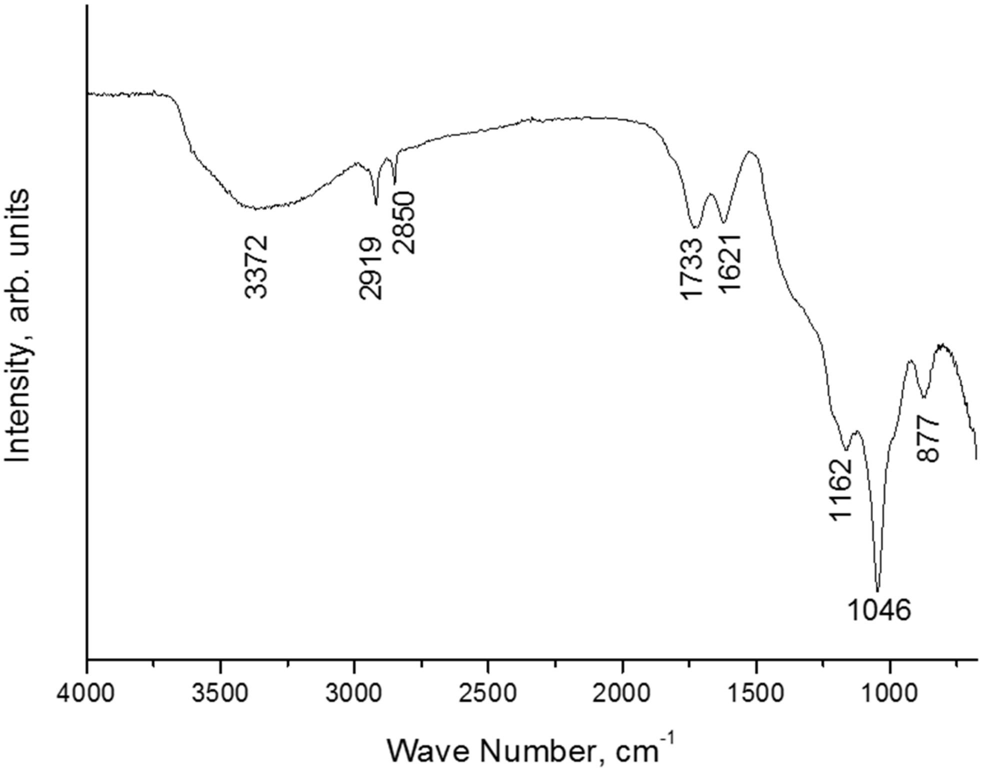

Figure S1. The IR spectrum of GO.

Table S1. Positions of the main peaks in the IR spectrum of soy wax.

Peak	X (cm^{-1})	Y (%T)
1	3307.55	95.34
2	2954.98	81.64
3	2915.08	41.01
4	2849.66	56.18
5	1736.99	53.13
6	1729.21	53.94
7	1470.89	71.50
8	1416.63	87.42
9	1391.26	79.48
10	1331.82	88.00
11	1286.40	81.95
12	1267.99	77.18
13	1255.42	76.36
14	1215.00	77.58
15	1194.84	70.65
16	1174.28	49.04
17	1105.97	74.73
18	1061.48	82.59
19	1047.55	82.76
20	1015.44	88.60
21	992.30	86.94
22	963.85	78.36
23	891.54	90.98
24	717.93	69.61
25	629.42	91.72
26	464.38	92.62
